# Association between exercise and clinical outcomes in patients treated with immunotherapy for solid tumors

**DOI:** 10.3389/fimmu.2025.1694045

**Published:** 2025-10-31

**Authors:** Kimberly Loo, Jessica A. Lavery, Jessica Palmer, Winston Guo, Whitney P. Underwood, Chaya S. Moskowitz, Lee W. Jones, Allison S. Betof

**Affiliations:** ^1^ Department of Oncology, Weill Cornell Medicine, New York, NY, United States; ^2^ Department of Epidemiology and Biostatistics, Memorial Sloan Kettering Cancer Center, New York, NY, United States; ^3^ Department of Medicine, Memorial Sloan Kettering Cancer Center, New York, NY, United States; ^4^ Department of Oncology, Stanford University School of Medicine, Stanford, CA, United States

**Keywords:** exercise oncology, immunotherapy response, immune check inhibitor (ICI), solid tumor, tumor mutation burden

## Abstract

Immune checkpoint inhibitors (ICI) have revolutionized the treatment of advanced cancers, but overall response rates remain modest and adjunct therapies to enhance efficacy of ICI are of great interest. This retrospective study examines the association between exercise and clinical outcomes in 258 patients with advanced solid tumors receiving ICI. The results suggest an association between exercise and better clinical outcomes, particularly in patients with high tumor mutation burden, though improvements in clinical benefit rate (58% vs. 51% for exercisers and non-exercisers, respectively) and one-year overall survival (67% vs. 58% for exercisers and non-exercisers, respectively) are not statistically significant. Our discovery-based findings in conjunction with preclinical evidence create a strong rationale for translational studies to formally investigate the effects of structured exercise therapy in combination with ICI in patients with solid tumors.

## Introduction

Use of immune checkpoint inhibitors (ICI) has revolutionized cancer care and formed a new pillar of cancer treatment with potential for durable control of advanced cancer. Despite tremendous successes with ICI, overall response rates across solid tumors remain modest, creating an unmet need for predictive factors of response. Identifying adjunct, low toxicity, combination strategies to augment response to ICI is an area of intense investigation. Host factors such as genetic predisposition, diet, and body mass index (BMI) contribute to and/or modify the antitumor efficacy of ICI (1). BMI predicts response to immune checkpoint inhibitor therapy in multiple tumor types (2). As another modifiable host factor, the role of exercise has been less well-delineated. Preclinical studies demonstrate exercise, a potent regulator of host physiology, promotes anti-tumor immunity in solid tumors that, when combined with ICI, enhances tumor suppressive activity (3, 4). In patients with cancer, exercise decreases circulating myeloid cells and increases circulating NK cell number and cytotoxic function (5). Preliminary data suggest that exercise may augments response to ICI in patients with cancer but further research is needed (6). Clinical translation of whether exercise improves response to ICI in patients with solid tumors has received minimal attention. Accordingly, we examined the impact of exercise on clinical outcomes in patients receiving ICI for solid tumors.

## Methods

We performed a retrospective analysis of 258 patients with solid tumor malignancies receiving ICI regimens for advanced, unresectable disease with annotation of exercise within one year prior to ICI initiation at Memorial Sloan Kettering Cancer Center (**Table 1**). Patients receiving adjuvant ICI were excluded. Best overall response (BOR) of complete response (CR), partial response (PR), stable disease (SD), or progressive disease (PD) to ICI-containing regimen were assessed based on clinician determination of response and review of imaging. Clinical benefit (CR, PR, SD) by exercise status was evaluated using logistic regression, adjusting for sex and BMI. Overall survival (OS), defined as the time from first dose of ICI to death, was analyzed using Kaplan-Meier methods and modeled using Cox proportional hazards regression as a function of exercise status, sex and BMI.

**Table 1:** Patient characteristics.

Characteristic	Overall	Exerciser	Non-Exerciser
Patients – no. (%)	258 (100)	163 (63)	95 (37)
Female – no. (%)	151 (59%)	91 (56%)	60 (63%)
Age at regimen initiation in years – median (interquartile range)	67 (59, 74)	66 (58, 72)	72 (60, 77)
Body mass index – median (interquartile range)	25.4 (22.5, 29.4)	26.1 (22.2, 31.3)	25.1 (22.6, 29.1)
Race – no. (%)			
White/Caucasian	222 (86)	145 (89)	77 (81)
Asian	12 (4.7)	6 (3.7)	6 (6.3)
Black/African American	14 (5.4)	8 (4.9)	6 (6.3)
Other	5 (1.9)	2 (1.2)	3 (3.2)
Missing	5 (1.9)	2 (1.2)	3 (3.2)
Histology – no. (%)			
Lung	83 (32)	46 (28)	37 (39)
Melanoma	21 (8.1)	13 (8.0)	8 (8.4)
Kidney	21 (8.1)	11 (6.7)	10 (11)
Breast	19 (7.4)	12 (7.4)	7 (7.4)
Endometrial	19 (7.4)	16 (9.8)	3 (3.2)
Ovarian	12 (4.7)	9 (5.5)	3 (3.2)
Sarcoma/Liposarcoma	12 (4.7)	8 (4.9)	4 (4.2)
Bladder/Urothelial	10 (3.9)	3 (1.8)	7 (7.4)
Esophageal, gastric	9 (3.5)	8 (4.9)	1 (1.1)
Other(1)	52 (20)	37 (23)	15 (16)
Checkpoint inhibition regimen – no. (%)			
Anti-CTLA-4	22 (8.5)	16 (9.8)	6 (6.3)
Anti-PD1/PD-L1	231 (90)	145 (89)	86 (91)
Anti-CTLA-4 and anti-PD-1	5 (1.9)	2 (1.2)	3 (3.2)
Tumor mutational burden (mt/Mb) available – no. (%)	226 (88)	71 (75)	155 (95)
Tumor mutational burden (mt/Mb) – median (interquartile range)	5 (3, 10)	7 (4, 11)	4 (2, 9)
Years from survey to IO initiation – median (interquartile range)	1.0 (0.5, 1.7)	1.0 (0.5, 1.6)	1.1 (0.5, 1.8)

^1^Head and neck (n=6), colorectal (n=7), thyroid (n=5), prostate (n=5), pancreas (n=4), uterine (n=3), cervix (n=3), adrenocortical (n=2); hepatocellular (n=2), mesothelioma (n=2), cutaneous squamous cell carcinoma (n=2), melanoma (n=2), GE junction adenocarcinoma (n=1), glioblastoma (n=1), liver (n=1), Oligodendroglioma (n=1), pancreas neuroendocrine (n=1), head and neck adenoid cystic carcinoma (n=1), signet ring adenoncarcinoma of the GEJ (n=1), merkel cell (n=1), and appendix (n=1).

### Exercise assessment

Exercise was assessed using a validated survey and defined as any moderate or strenuous exercise per week; non-exercisers were defined as no moderate or strenuous exercise. Exercise history was prospectively evaluated using the Godin Leisure Time Exercise Questionnaire (GLTEQ) (7). The GLTEQ contains three questions that assess the average frequency of mild, moderate, and strenuous-intensity exercise sessions of at least 15 mins/session in a typical 7-day period during leisure-time. Participants also reported the average duration of exercise within each intensity category. The frequency of sessions per week within each intensity category was multiplied by the average duration to calculate exercise minutes per week in each intensity category, which was then summed for calculation of total minutes of exercise per week. Non-exercise was defined as 0 minutes of moderate or vigorous intensity exercise per week and exercise >0 minutes of moderate or vigorous intensity exercise per week.

### TMB assessment

Tumor genomic sequencing data (8) was available on 226 patients (88%), enabling analysis of whether tumor mutational burden (TMB), an established predictor of ICI response, influenced clinical response to ICI and exercise, assessed *via* interaction terms in the models. Patients entered the risk set at the time of genomic sequencing in OS analyses by TMB to avoid introducing a delayed entry bias. Low TMB defined as <10 mt/Mb, high TMB defined as ≥10 mt/Mb.

## Results

**Figure 1:**
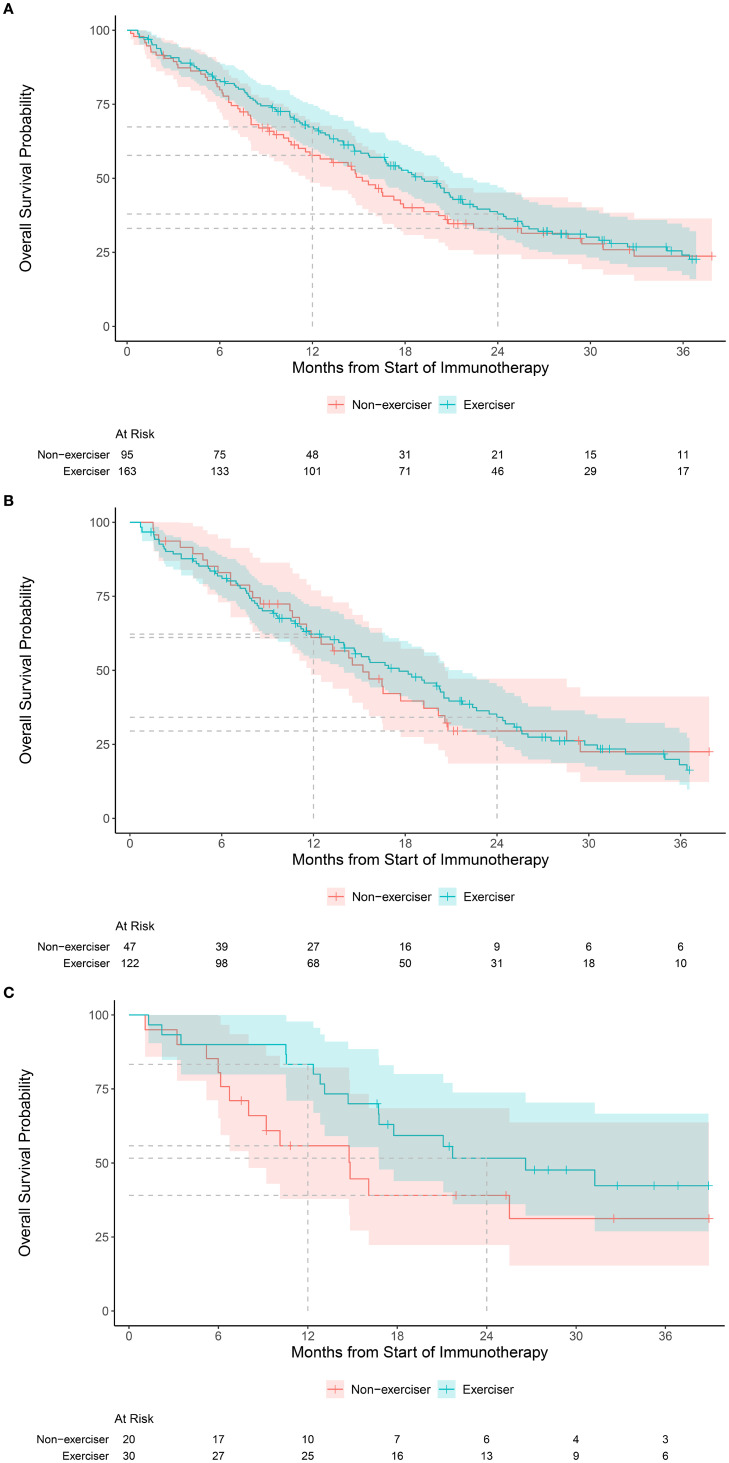
Kaplan-Meier estimates of overall survival in the: **(A)** overall cohort according to exercise status, categorized as exercisers (any moderate or vigorous exercise per week) versus non-exercise (no moderate or vigorous exercise per week), **(B)** Kaplan-Meier estimates of overall survival according to exercise status for tumors with low (<10 mt/mb) mutational burden, and **(C)** Kaplan-Meier estimates of overall survival according to exercise status for tumors with high (≥10 mt/mb) mutational burden.

The clinical benefit rate was 58% for exercisers and 51% for non-exercisers (odds ratio (OR), 1.37, 95% CI, 0.82, 2.29). Median follow-up was 1.8 years (interquartile range 1.1, 2.7) among patients alive at the end of the study. During follow-up, a total of 181 deaths were observed. One year OS was 67% (95% CI, 60, 75) for exercisers and 58% (95% CI, 48, 69) for non-exercisers; two year OS was 38% (95% CI, 31, 47) for exercisers and 33% (95% CI, 24, 45) for non-exercisers (hazard ratio [HR], 0.87, 95% CI, 0.64, 1.19; **Figure 1A**).

The rate of clinical benefit for low (<10 mt/mb) TMB (n=174) was 55% for exercisers and non-exercisers; for high ( ≥ 10 mt/mb) TMB (n=52) was 73% for exercisers and 55% for non-exercisers. One year OS for patients with low TMB was 62% (95% CI, 54, 72) for exercisers and 61% (95% CI, 48, 77) for non-exercisers (**Figure 1B**); for high TMB, one year OS was 83% (95% CI, 71, 98) for exercisers and 56% (95% CI, 38, 82) for non-exercisers (**Figure 1C**). Interaction terms between TMB and exercise were not statistically significant for clinical benefit or OS.

## Discussion

Identifying modifiable host factors that can influence the efficacy of ICI-based regimen is of great interest. Preclinical models indicate that exercise retards tumor growth and enhances the efficacy of ICIs (9–15). Multiple mechanisms have been proposed including augmentation of inflammation and immune infiltration tumor microenvironment (e.g. making tumors “hot”) (11, 13, 16), enhanced cytokine signaling mediating CD8+ T-cell and NK cell dependent cytotoxicity (10, 14), and alteration of the gut microbiome promoting accumulation of metabolites that augment the efficacy of ICI via CD8+ T cells (15). Translating these findings to clinical benefit for patients receiving ICIs is of the utmost urgency.

This single-center cohort study suggests that exercise may be associated with better clinical outcomes in patients receiving ICI for advanced solid tumors, although results were not statistically significant. Our results indicate that for patients with high tumor mutation burdens may be more likely to elicit an effect exercise-immune response and improved overall survival. This is consistent with prior work demonstrating that moderate and high levels of physical activity were associated with prolonged survival following ICI treatment in ICI responsive tumors (melanoma, Non small cell lung cancer (NSCLC), Renal Cell Carcinoma (RCC)), including those treated in the adjuvant setting (4).

Study limitations include small sample size, observational retrospective design, reliance on self-reported exercise, and restriction to survivors who were alive and willing to complete an exercise survey after initial cancer diagnosis. Moreover, objective measures of exercise capacity were not available at baseline or follow-up, which is a known prognostic factor across cancer entities (17). The lack of information on exercise type (endurance, resistance, or balance training), degree of supervision, and intensity further limits interpretation, underscoring the need for prospective studies with structured exercise phenotyping. In addition, the inclusion of different cancer entities may obscure disease-specific effects of exercise, as exercise interventions may need to be tailored by tumor type. Lung cancer patients, which represents the majority of our cohort, typically carry higher cardiovascular risk and ventilatory limitations, which may influence exercise, participation, and subsequent outcomes. Prior evidence has demonstrated that even small amounts of exercise may reduce mortality in cancer patients and improve the quality of life, fatigue, and exercise capacity (18, 19). Future studies examining exercise by unique tumor types with associated baseline cardiovascular risk factors and impact on exercise fitness is needed. Finally, cardiovascular comorbidities and acute-ICI related events (e.g. myocarditis, pneumonitis) were not fully captured, yet are important determinants of survival in this population. Larger retrospective or prospective studies are needed to increase statistical power, clarify these associations, and validate our exploratory findings.

Notwithstanding these limitations, our discovery-based findings in conjunction with existing preclinical evidence create a strong rationale for translational studies to formally investigate the effects of structured exercise therapy in combination with ICI in patients with solid tumors. Our work extends on these findings by including TMB analysis, demonstrating that another prognostic factor to ICI in conjunction with exercise associates with improved OS to ICI treatment among patients with unresectable or metastatic solid tumors, and highlighting a hypothesis-generating association that warrants confirmation in prospective studies. Prospective trials examining exercise physiology of patients undergoing ICI treatment are ongoing (NCT06026111, NCT06983899, NCT06672120). Taken together, our study provides a strong rationale for ongoing studies to investigate the effects of integrating structured exercise therapy with immunotherapy strategies in patients with advanced solid tumors to improve clinical outcomes. Our intriguing molecular findings support a novel hypothesis that tumors with a high number of clonal neoantigens propagated by high TMB may be more likely to elicit effective exercise-induced immune response.

## Data Availability

The raw data supporting the conclusions of this article will be made available by the authors, without undue reservation.
